# Land cover type modulates the distribution of litter in a Nordic cultural landscape

**DOI:** 10.1371/journal.pone.0275463

**Published:** 2022-11-09

**Authors:** Sara K. K. Eide, Linn N. Leh, Katinka S. Eines, Ingunn Hovland, Marit By, Elise W. Ingvaldsen, Marthe Tinlund, Evan Emerita, Luciano A. Machado, Stian Brønner, Erlend B. Nilsen, Sam M. J. G. Steyaert

**Affiliations:** 1 Faculty of Biosciences and Aquaculture, Nord University, Steinkjer, Norway; 2 Norwegian Institute for Nature Research, Trondheim, Norway; Northeastern University (Shenyang China), CHINA

## Abstract

Litter pollution is a global environmental problem that occurs in virtually all ecosystems. Scientific research on anthropogenic litter and its environmental impacts focusses predominantly on plastics and the marine environment. Little empirical knowledge exists about the distribution and ecological impacts of litter in terrestrial environments, where most litter is produced. To start closing that knowledge gap, we investigated the distribution of litter in a cultural landscape in central Norway and in relation to land cover types. We registered and collected litter in 110 survey plots that were randomly stratified across various land cover types. Our results show that land cover type modulates the occurrence, abundance, fragments size, and that litter is most present and abundant in or near land cover types associated with high human activities. Plastic was by far the most common litter material type, although the litter community (in terms of materials type) was not independent from land cover type. This knowledge can help to inform and optimize litter management and clean-up activities in terrestrial landscapes. How and to what extent the spatial structure of the litter community mediates ecological effects across various land cover types remains unknown to a large extent and warrants further study.

## Introduction

The distribution and dispersal of anthropogenic litter (hereafter litter) in the environment is a global problem. Depending on the size and nature of litter, it can affect all levels of the ecological hierarchy: from the subcellular level to entire ecosystems and biomes [1–5]. For example, chemical leaching of plastics can induce DNA mutations in organisms [6], macroparticles can injure or entangle wildlife [7], or suffocate, intoxicate, and starve organisms when ingested [8]. Furthermore, accumulation of litter in oceans can cover vast areas (e.g., the Great Atlantic Garbage Patch, covering about 1.6 million km^2^) that may impair marine ecosystem functioning [9]. Litter also has economic impacts, as it can hamper commercial activities such as fishing and tourism, and because litter management and clean-up actions can be expensive [10].

The vast majority of studies that investigate litter and its environmental impacts have been conducted in marine environments [3, 11]. The marine environment is known as a ‘sink’ habitat where much litter eventually ends up, for example, about 96% of all microplastic studies focus on oceans and coastal ecosystems [12]. Very little knowledge exists about the origin, distribution and dispersal, and ecological impacts of litter (especially plastics) on land, where virtually all litter is produced [11–13]. However, identifying origins and transport pathways of litter dispersal in terrestrial systems is crucial for managing and mitigation of this type of pollution [3, 11, 13–15], but has been largely overlooked in the literature [11].

Litter implies solid waste made or used by humans (e.g., plastic bags, cans, etc.) and discarded or lost in inappropriate locations [16, 17]. It is therefore reasonable to assume that litter distribution and abundance in terrestrial environments are tied to human population densities or activities [3, 14, 18].

One could expect that litter particles are more abundant in cities and villages compared to more natural habitats like forests; or that the occurrence of litter shows a distant decay relationship with core areas of human activities or sink habitats. In addition, specific terrain features such as edges between open terrain and vegetation, water and land, and roadside verges may be more prone for littering or litter accumulation during dispersal [15, 16], and are thus likely to increase the variation in the distribution of litter across the landscape. Due to the inherent association between litter and human activity, one could also expect variation in the composition of different litter types (the ‘litter community’) and particle sizes across land cover types or along spatial gradients (e.g., distance to roads or urban areas) [19, 20]. This is important, because it implies that ecological impacts induced by litter can vary across the landscape [2]. Furthermore, urbanization has reached unprecedented levels [21] and leads to increased human inference into natural ecosystems [18], which most likely also promotes litter distribution in such areas. Better understanding the link between land cover type and litter distribution may therefore be valuable to anticipate of environmental impacts of urbanization.

In this study, we assessed the distribution of terrestrial litter in relation to land cover types in a cultural landscape in central Norway. We expected that (Hypothesis 1, H1) the distribution of litter is not uniform across land cover types, with both the litter detection probability (H1a) and abundance (H1b) being highest in land cover types associated with high levels of human activity (e.g., urban areas, roadside verges) and sink habitat (e.g., beaches) and lowest in more natural habitat types such as forests. Furthermore, we expected that (H1c) the litter detection probability would decrease with distance to roads, i.e., linear features with concentrated human activity, and that (H1d) litter fragment size would be largest in land cover types associated with litter sources (e.g., urban areas, agricultural land) compared to sink habitats (e.g., beaches).

In addition, we expected that (Hypothesis 2, H2) the composition of the litter community in terms material type would vary among land cover types, with, for example, metal scrap (e.g., discarded machinery) being more related to agricultural land and its edges, and plastics being most common at sink habitat (e.g., beaches). Our research sheds light on the distribution and composition of litter in terrestrial ecosystems, which has been largely overlooked in the literature [11, 22], and has important implications for litter accountability and management.

## Methods

### Study area

**Fig 1:**
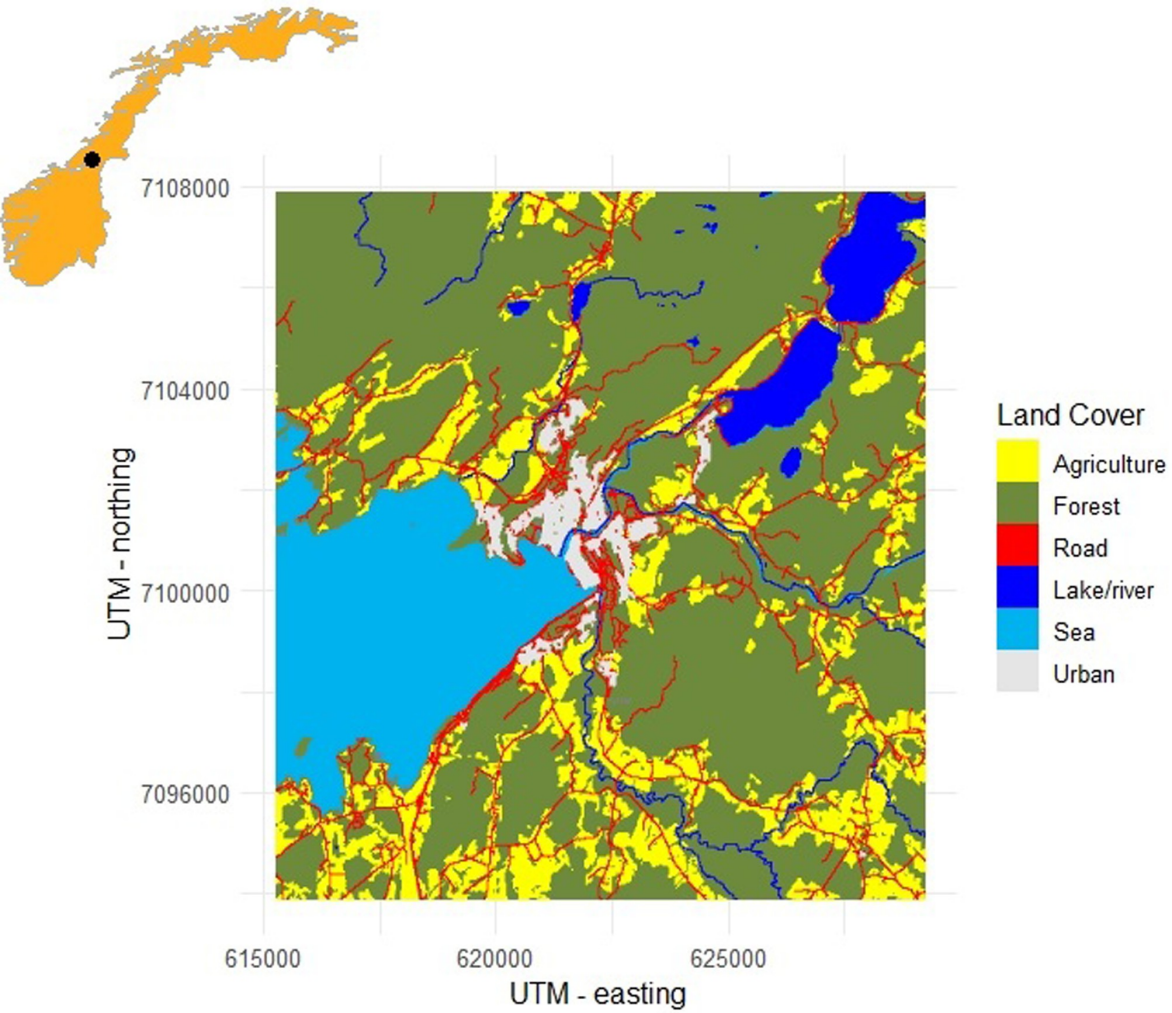
A map of the study area. In the upper left corner is a map of Norway with the study area in Steinkjer is marked in black. Underneath is an overview map of the different land cover types in our study area as categorized in this study.

We evaluated our hypotheses in Steinkjer municipality in Trøndelag county, central Norway. Steinkjer is located in the innermost part of the Trondheimsfjorden (64°N, 11°E) and lies in the south boreal zone (Fig 1). Steinkjer has an oceanic climate with an annual average temperature between 4–6°C and annual precipitation between 800- and 1500-mm. Snow cover typically lasts for about 3–5 months [23]. The topography of the municipality is characterized by low hills and wide valleys that run in a southwest-northeast direction. The municipality has a population of about 24000 inhabitants (13 inhabitants/km^2^) [24] and covers approximately 2100 km^2^ [24]. Steinkjer has a cultural landscape with a matrix of agricultural and forest land surrounding small settlements or villages. Forest lands comprise commercial and natural coniferous forest, and are intersected by bogs, rivers, and lakes [24]. Roads and buildings cover 8 and 3.5 km^2^, respectively [24]. We defined the operational study area as a square of 196 km^2^ with Nord University campus Steinkjer as the midpoint (N: 622235 E:7100896, WGS84 UTM Z32).

### Land cover variables

We used the ‘SatVeg’ raster dataset (Johansen 2009), and the ‘NVE_Elvenett’ [25] and ‘Vbase’ [26] vector based datasets to produce a land cover classification map that contained the following land cover classes: ‘forest’, ‘ocean’, ‘agriculture’, ‘urban’, ‘river’, ‘lake’, ‘road’, ‘forest/agriculture edge’ (hereafter ‘edge’), ‘beach’, ‘lake shore’, and ‘unclassified’. The three datasets were assigned the WGS84 UTM zone 32 coordinate system. The NVE_elvenet and Vbase datasets were rasterized and snapped into 30 x 30m rasters to match the SatVeg raster.

The SatVeg dataset is a nationwide land cover map based on image classification of Landsat 5 TM and Landsat 7 ETM+ satellite imagery and includes 25 vegetation types. The dataset was produced in 2009 by the Northern Research Institute NORUT [27]. We reclassified the SatVeg data to obtain the land cover classes ‘forest’, ‘water’, ‘agriculture’, ‘urban’ and ‘unclassified’. We updated that raster with NVE_Elvenett to classify ‘water’ into ‘rivers’ and ‘lakes’. The NVE_Elvenett data is a vector-based river network dataset and was produced in 2002 by the Norwegian Water Resources and Energy Directorate [25]. The Vbase dataset is a continuously updated vector-based and nationwide dataset that contains all drivable roads > 50m and distinguishes between different road types [26]. We merged all the road types into one category, which we used for updating our land cover classification. Within the land cover classification, we selected boundaries between forest and agriculture, ocean and land, and lakes and land and reclassified those into ‘edge’, ‘beach’, and ‘lakeshore’, respectively. Furthermore, we used the Vbase dataset to calculate the Euclidean distance to the nearest road (irrespective of road class) for all 30 x 30 raster cells in the study area. Geoprocessing was conducted in R software [28], using the ‘raster’ [29], ‘rgeos’ [30], ‘rgdal’ [31], ‘sp’ [32], ‘mapview’[33] and ‘maptools’ [34] packages. Please refer to S1A in S1 Appendix for details about all geoprocessing.

### Data collection

We used stratified random sampling to distribute 30 survey plot locations in all strata (except for ‘sea’, ‘lake’, and ‘unclassified’) in the operational study area and randomly assigned two cardinal directions for each plot. We used handheld GPS units (Garmin GPSmap 66s) to navigate to the exact plot location in continuous habitat (e.g., agriculture, forest) or as close as possible to the plot location for more discrete habitat types (e.g., roadside verges, lakeshores, and edge habitat). There, we laid out a 50 m rope in the first predefined cardinal direction (in continuous habitat), or along linear features (e.g., riverbanks and roadsides) following the first predefined cardinal direction as close as possible. In cases where sampling was not possible following the first or second predefined cardinal direction (e.g., transects crossing habitat types), we selected a direction as close as possible to the second predefined cardinal direction. We walked along the rope and registered all visible litter within one meter from each side of the rope. We collected all litter in plastic bags for further registration, except for litter that was too large to collect (e.g., old machinery) or unsanitary. These items were identified and measured at the site. All field work was carried out by teams of minimum two field workers, using sanitary gloves when considered needed for litter collection. Fieldworkers were calibrated during two test trials, of which one is included in the dataset. We aimed to sample as many survey plots as possible, and in a balanced manner regarding the number of plots in each strata. All fieldwork was conducted between 5^th^ and 9^th^ of October 2020.

The collected litter was labelled with a unique identifier in the lab, assigned a primary material type (plastic, paper, metal, glass, clay, paint, wood, other composite) and, if possible, an originally intended use (e.g., food wrapper, tobacco pouch; referred to as ‘origin’) S1B in S1 Appendix to the best of our knowledge and internet searches. Every item was measured in length and width (and depth if possible) and archived.

### Data analysis

#### Hypothesis 1—Distribution of litter in relation to land cover type

We used generalized linear models (GLM) with a binomial distribution to evaluate the relationship between litter detection in a survey plot (yes = 1, no = 0) and land cover type (H1a). We formulated two candidate models for this, i.e., one that included land cover type as an explanatory variable (the ‘land cover model’), and a null model. We used the Akaike Information Criterion corrected for small sample sizes (AICc) to contrast those two models. We considered the land cover model as informative if the null model had an AIC difference value (ΔAICc) > 2 [35, 36]. We repeated this procedure to evaluate the relationship between litter abundance in survey plots and land cover type (H1b), using a negative binomial GLM. We opted for a negative binomial GLM, because a preliminary GLM with a Poisson distribution showed considerable overdispersion (dispersion statistic: 49.06).

We assessed if litter detection probability decreased with proximity to roads (H1c) using a binomial GLM. We subsetted the data to exclude land cover types ‘urban’ and ‘roadside verges’ because of inherent collinearity with proximity to roads. We used litter detection in a survey plot (yes = 1, no = 0) as the response variables, and land cover type and distance to the nearest road as explanatory variables. We constructed 5 candidate models: an interaction and additive model based on both explanatory variables, two models with the explanatory variables as single effects, and a null model. We considered the model with the simplest structure within a ΔAICc range of 0–2 as the most parsimonious model and considered models with a ΔAICc value > 2 as inconclusive [35, 36]. For the binomial models, we evaluated model fit by calculating the ratio between the residual deviance and the residual degrees of freedom and considered values between 0.8 and 1.5 as acceptable [37].

Because of repeated observations per survey plot, we used linear mixed effects regression models to assess the relationship between litter particle size (the largest dimension measured) and land cover type (H1d). We log-transformed the response variable ‘particle size’ and included survey plot ID as random effect on the intercept. We used the same model selection procedure as described earlier. Following Zuur et al. (2009), we used maximum likelihood estimation for model selection, and fitted the model using restricted maximum likelihood estimation for interpretation. We visually assessed model fit using residual plots [37]. We used the lme4 package [38] for fitting the mixed effects models, the AER package [39] for calculating dispersion statistics for Poisson GLMs, and the MuMIn package [40] for model selection.

#### Hypothesis 2—The litter community

Wordcloud analysis entails a qualitative and visual approach to analyze the relative frequency of occurrence of specific words or phrases in datasets or literature. The analysis results in a cloud visualization of words or phrases, in which their font size indicates their relative abundance in the dataset [41]. Wordclouds facilitate content analysis and expand reader comprehension [41]. We used wordclouds to illustrate how the composition of litter in terms of origin and material varied among land cover types using the ggwordcloud package [42]. In addition, we used a chi-square test for potential differences in the litter community according to material and land cover types. We merged relatively rare material types such as wood, clay, paint, glass into material type ‘other’ to avoid cells with frequencies lower than 5 in cross tables [43]. We did not perform such a test on the material origin because of many singularities in the data. All data and R code for the statistical analyses is available as S1B and S1C in S1 Appendix, respectively.

## Results

We surveyed 110 plots for litter (N agriculture = 16, N urban = 11, N forest = 14, N edge = 13, N beach = 11, N lakeshore = 14, N river = 13, N road = 18). We detected litter in 63 plots (i.e., 57% of all plots). In plots with litter, the number of litter items ranged between 1 and 158, and totaled 932 (S1B in S1 Appendix).

### Hypothesis 1—Spatial distribution of litter in relation to land cover type

**Fig 2:**
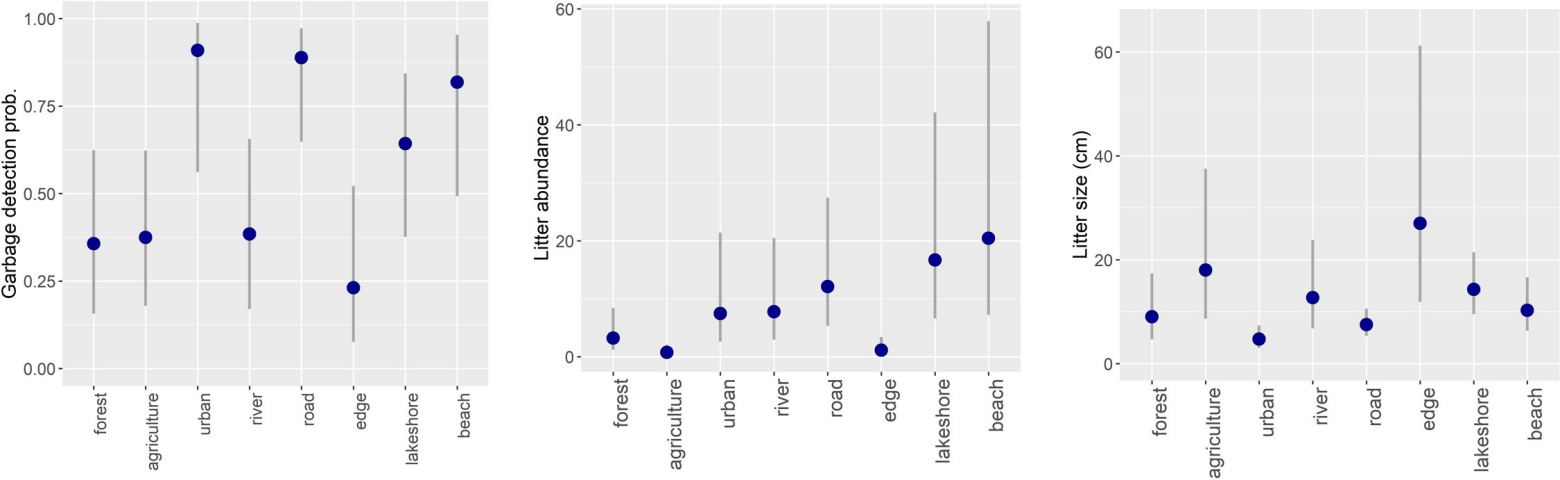
Predicted litter detection probability (H1a, upper panel), abundance (H1b, mid panel), and fragment size (H1d, lower panel) in 100 m^2^ sampling transects (50 x 2 m) in relation to land cover classes in the cultural landscape of Steinkjer municipality, central Norway. Fieldwork was carried out during autumn 2020. Blue dots indicate predicted means, and whiskers indicate 95% confidence intervals.

The model that contained land cover type (ΔAICc = 0.00) outperformed the null model (ΔAICc = 16.04) for detecting litter in survey plots (H1a). The predicted litter detection probability was highest along roads (mean: 88.9%, 95% confidence interval CI: 64.8–97.2%) and in urban areas (mean: 90.9%, CI: 56.2–98.7%), and lowest in edge land cover types (mean: 23.1%, CI: 7.6–52.2%). The predicted 95% confidence interval was largest for riversides (mean: 38.5%, CI: 17.0–65.6%, CI range: 48.6%) and lowest for roadside verges (CI range: 32.4%) (Fig 2, upper panel). The selected model had a dispersion statistic of 1.16. For model selection diagnostics, parameter estimates, predicted values, refer to S1–S3 Tables.

The model that contained land cover type (ΔAICc = 0.00) outperformed the null model (ΔAICc = 12.51) for predicting litter abundance in survey plots (H1b). Litter was most abundant at beaches (mean: 20.5 items, CI: 7.2–57.9), lakeshores (mean: 16.7 items, CI: 6.6–42.1), and roadside verges (mean: 12.1 items, CI: 5.3–27.4), whereas it was least abundant in agricultural areas (mean: 0.8 items, CI: 0.3–2.1). Variation in litter abundance was particularly large for beaches (CI range: 50.7), lakeshores (CI range: 35.5) and roads (CI range: 22.1) (Fig 2, mid panel). For model selection diagnostics, parameter estimates, and predicted values, refer to S1, S4 and S5 Tables.

**Fig 3:**
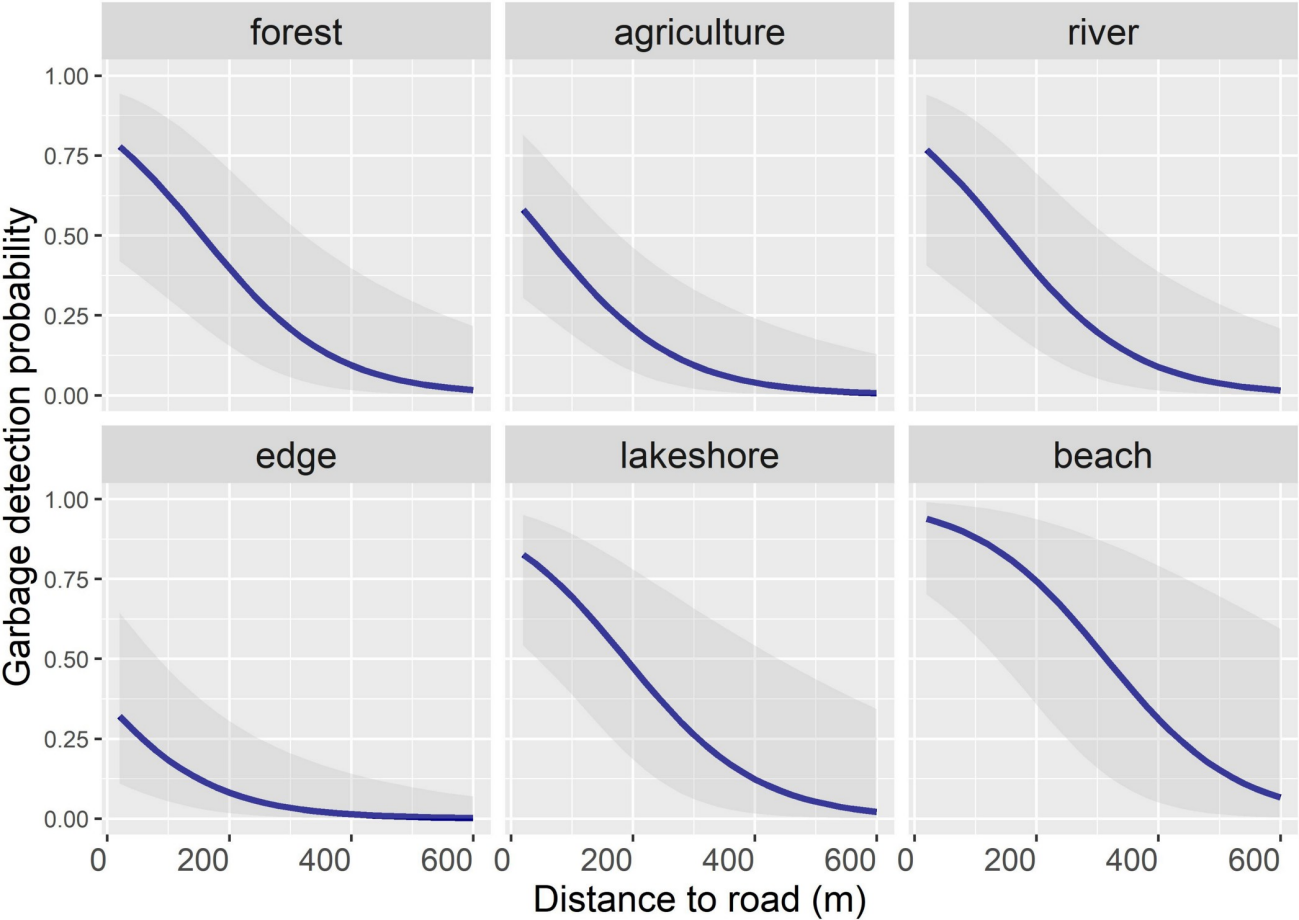
Predicted litter detection probability in 100 m^2^ sampling transects (50 x 2 m) in relation to distance to the nearest road (m) and according to land cover classes in the cultural landscape of Steinkjer municipality, central Norway (H1c). Fieldwork was carried out during autumn 2020. The grey polygons indicate the 95% confidence region around the predicted means (blue lines).

The model that included distance to the nearest road and land cover type as additive terms performed best to assess litter detection probability in the landscape (H1c). All other candidate models were considered inconclusive (ΔAICc ≥ 3.43). Litter detection probability rapidly decreased with increasing distance to the nearest road. Estimated detection probabilities approached 0 in all land cover types at distances of about 400–600 m to the nearest road, but the uncertainty around the estimates varied largely among land cover types (Fig 3). Uncertainty was largest for beaches (estimate β = 2.4, standard error se = 1.01), whereas it was relatively low for edge habitat (β = -1.08, se = 0.88) (Fig 3). The ratio between the residual deviance and the residual degrees of freedom of the selected model was 1.14. We refer to S1 and S6 Tables for model selection diagnostics and parameter estimates, respectively.

The model that contained land cover type (ΔAICc = 0.00) outperformed the null model (ΔAICc = 9.78) for evaluating if litter fragment size differed across land cover types (H1d). The largest litter fragments were found along edges (predicted mean: 27.0 cm, CI: 11.9–61.2) and on agricultural fields (mean: 18.0 cm, CI: 8.7–37.5), whereas smallest fragments occurred in urban land cover types (mean: 4.7 cm, CI: 3.0–7.3) and along roads (mean: 7.5 cm, CI: 5.4–10.6). Variation in litter size was also particularly large along edges (CI range: 49.3 cm) and was smallest in urban areas (CI range: 4.3 cm) (Fig 2, lower panel). Refer to S1, S7 and S8 Tables for model selection diagnostics, parameter estimates, and predicted values, respectively. Plotting the model residuals versus the fitted values suggested that no model assumptions were violated (S1 Fig).

### Hypothesis 2 –The litter community

**Fig 4:**
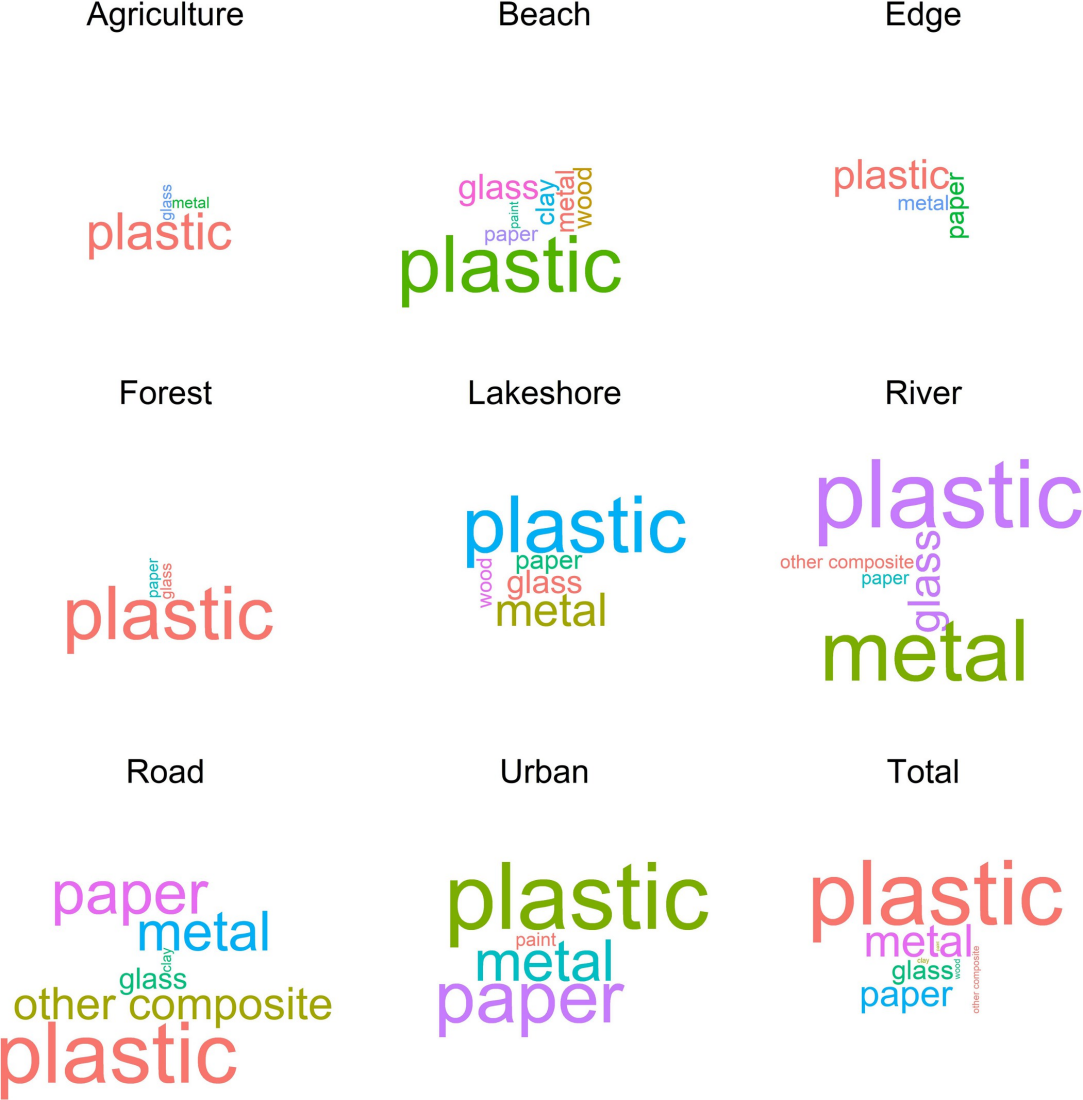
Wordcloud the material type of all registered litter items distributed by land cover type and in total. Litter items were registered in 110 sampling transects (50 x 2 m) distributed in a cultural landscape of central Norway during autumn 2020. Material types in the wordcloud are scaled in size relative to their abundance per land cover type and in total. Note that relatively rare material types were condensed into class ‘other’ to perform the formal chi-square test.

A chi-squared test revealed that some litter material types occurred substantially more frequently or less frequently than expected in certain land cover types (χ^2^ = 210.83, p = <0.001, 21 degrees of freedom, N = 932). For example, plastics occurred more frequently (observed O = 207) on beaches than expected (expected E = 180.8), and metal scrap was observed (O = 36) more frequently than expected (E = 9.1) at river sides. Paper items occurred more frequently than expected in urban areas (O = 19, E = 5.1) and along roads (O = 29, E = 13.6). Overall, plastic was by far the most commonlitter material (O = 749) in all land cover types, followed by metal (O84), paper (O = 58), and other (O = 41) (Fig 4 and S9 Table). The litter community in terms of its original intended use appeared similarly diverse at beaches, lakeshores, riversides, roadside verges, and urban areas (S2 Fig).

## Discussion

Our results show that the distribution of litter in a terrestrial agricultural landscape was structured according to land cover type and human activity (i.e., roads). We found higher litter detection probabilities (H1a) and abundances (H1b) in land cover types that were closely associated with human activity (i.e., urban, roads), as well as in sink habitats (i.e., beaches, lakeshores), and that litter detection rates decreased with increased distance to roads (H1c). Furthermore, litter fragment size varied among land cover types (H1d), with the largest fragments being found in edge land cover types and the smallest items along roads and in urban areas. We also found that material type of litter was not independent of land cover type (H2), although plastic items were overall by far the most prevalent. Undoubtedly, we underestimated litter occurrence and abundance, and overestimated fragment size in all land cover types. This is because we visually assessed litter presence and abundance and could only measure fragment size of detected items. Especially smaller items in vegetated land cover types are likely to remain undetected.

Litter is inherently linked to humans and human activities, but also spreads into places where human activity is low (e.g., wilderness areas or marine reserves) [13]. Not surprisingly, we found that litter occurrence and abundance was highest in urban environments and near roads, where the majority of litter is produced by both consumers and producers of goods (manufacturers and industries) [4]. Furthermore, littering behavior also appears to be stimulated in already littered environments compared to ‘clean’ environments [16]. This may make urban areas and roadside verges particularly prone to tumbling into a positive feedback loop of litter accumulation and littering. In line with our expectation, litter occurrence and abundances were also high at land cover types considered as litter ‘sinks’: beaches and lake shores. Surprisingly, litter detection probability was lowest at edges between land cover types. We expected that such edges would function as mesh for ‘capturing’ or holding litter, but this was not supported by our data.

The 95% confidence regions were particularly large and right skewed for predicting litter abundance at beaches, lake shores, and riversides. This is probably and partly explained by the overall low sample size of surveyed plots per land cover types, but also by the nature of the distribution of litter within the land cover types. For example, many survey plots at beaches, lakeshores, and riversides were relatively ‘clean’ and held none or few litter items, whereas these land cover types also contained litter ‘hotspots’, i.e., where litter has been traditionally dumped or naturally accumulated. For example, we discovered a litter dump site at a riverbank. This dump site extended way beyond the size of our survey plot and included metal containers, a bike, a metal gate, glass bottles, plastic fragments, tires, discarded machinery, fencing material, and spray cans. We discovered several additional dump sites during parallel fieldwork, and they often contain large amounts of plastic sheets, metal scrap, and highly toxic items such as car batteries (personal observations). Such dump sites can develop over several generations and are common in cultural landscapes in Norway [44, 45]. Very little is known, however, about how such dump sites impact their surrounding ecosystems.

Overall, litter item size was larger in land cover types with the lowest litter abundances, i.e., edges and agricultural land, whereas fragment size was relatively small in urban areas and roadside verges. This result was only partially in line with our expectations, as we predicted that litter fragment size would be largest in land cover types associated with the litter sources, like urban areas, roadside verges, and agriculture lands, compared to sinks habitats such as beaches. We indeed found that larger litter items occurred at agricultural fields (e.g., larger plastic fragments), but the urban areas and roadside verges were dominated by small items (e.g., snus pouches, cigarette filters, small plastic fragments). Edge habitat included edges between forest and agricultural and harbored the largest litter items, including discarded agricultural machinery and drainage pipes. We suggest that the dominance of smaller litter items in urban areas and along roads can be explained by two complementary mechanisms. First, the urban area is where humans consume and discard litter of relatively small size (e.g., cigarette butts, food wrappers, etc.), which is supported by our data (S1B in S1 Appendix). Secondly, human activity concentrates around urban areas and roads, which implies that litter items can easily become fragmented through physical abrasion by for example car tires or trampling [46].

As expected, the composition of litter in terms of material type varied among the different land cover types. Given a spatially structured litter community, it is likely to assume that also the ecological effects induced by litter can be spatially structured in the landscape. For example, cigarette butts and snus pouches were most common in urban areas. Urban birds commonly collect litter, including tobacco products, as nesting materials [47]. Even if used cigarette butts may be beneficial for nesting birds as a repellent against ectoparasites [47, 48], nicotine and heavy metals in tobacco products can also induce genotoxic damage in chicks and their parents [48]. Such tobacco-induced effects are unlikely to arise in remote habitats, where the likelihood of finding cigarette butts or snus pouches is much lower. Similarly, wildlife injuries and mortalities induced by discarded fishing gear are more likely to occur at for example beaches, lakeshores, and riversides [7, 49], whereas such effects should be rare in remote habitat. Since we found a close relationship between litter detection probability and proximity to roads, one could also expect that many ecological effects induced by litter (e.g., soil, ground, and surface water pollution) are most pronounced or have their origin near roads. However, little research has been conducted in relation to the distribution and differentiation of the litter community across land cover types in terrestrial ecosystems. Hence, how such spatial differentiation propagates into ecological effects across landscapes warrants further research [2].

Despite that the litter community was spatially structured across the landscape; plastic items were by far the most dominant in all investigated land cover types. Research on plastic pollution has long been focused on the largest sink: the oceans. However, there is a growing concern about plastic pollution in terrestrial environments [3, 4, 11, 12]. Rivers are acknowledged to be hotspots for the accumulation and transport of plastic pollution to the oceans [2, 4, 50]. Our results indicate that roads may also function as an important pathway for capturing, fragmenting, and dispersing plastics (and other litter types) towards sink habitat, because roadside verges had the highest litter detection probability of all surveyed land cover types, and plastics and smaller fragments dominated roadside verges. In addition, litter detection probability showed a strong distance decay relationship with proximity to roads, irrespective of land cover type. Smaller litter particles easily enter road drainage and sewage systems, further disintegrate into microparticles, and reach larger waterways and eventually the ocean [51].

## Conclusion

Our study shows that litter is omnipresent in a terrestrial cultural Nordic landscape, with estimated occurrences (per 100 m^2^) well above 80% in several land cover types. Forests, the most natural land cover type included in our study, even had an estimated mean litter occurrence of almost 36%. In the most contaminated land cover types, lakeshores, and beaches, roughly 1 litter item was predicted to occur per 4 square meters, with a maximum observed litter density of about 1.6 items/m^2^. Provided their omnipresence, we echo the need to increase the focus on litter pollution in terrestrial environments [3, 11, 12], both from a scientific, a governmental, and a lay perspective. For example, estimating litter abundance across the landscape using scientific methods can be beneficial to increase the efficiency of clean-up actions and to inform and educate the public about this environmental problem. Currently, several excellent litter cleanup initiatives exist (e.g., in Norway ‘Rydde’, ‘Plastjakten’, ‘Hold Norge Rent’), although they primarily focus on the marine environment. We suggest that such initiatives but especially also funding schemes extend their efforts towards terrestrial environments. Furthermore, the ecological impact of spatial differentiation of litter, including dump sites, remains largely unknown, and warrants further research.

## Supporting information

S1 Table Model selection diagnostics for the candidate models to evaluate H1a-d. Checkmarks (**✓**) indicate inclusion of specific model terms in candidate models. GLM = generalized linear model, LMM = linear mixed effects model, df = degrees of freedom, AICc = Akaike Information Criterion corrected for small sample sized, ΔAICc = the difference in AICc value between compared to the candidate model with the lowest AICc value, AICcw = AICc model weight.
(PDF)

S2 Table Output of the selected binomial logistic regression model to assess litter detection probabilities in 50 × 2 m plots (N = 110, surveyed in early October 2020) distributed across various land cover types in Steinkjer, Norway (H1a). The model including land-cover type outperformed the null model (ΔAICc = 16.04). β = estimate, se = standard error, z-value = test statistic, p-values < 0.05 are considered as statistically significant.
(PDF)

S3 Table Predicted litter detection probabilities and their 95% confidence intervals for 50 × 2 m plots (N = 110, surveyed in early October 2020) distributed across various land cover types in Steinkjer, Norway (H1a). se = standard error, LCL = lower 95% confidence limit, UCL = upper 95% confidence limit.
(PDF)

S4 Table Output of the selected negative binomial logistic regression model to assess litter abundance in 50 × 2 m plots (N = 110, surveyed in early October 2020) distributed across various land cover types in Steinkjer, Norway (H1b). The model including land-cover type outperformed the null model (ΔAICc = 12.51). β = estimate, se = standard error, z-value = test statistic, p-values < 0.05 are considered as statistically significant.
(PDF)

S5 Table Predicted litter abundance and their 95% confidence intervals for 50 × 2 m plots (N = 110, surveyed in early October 2020) distributed across various land cover types in Steinkjer, Norway (H1b). se = standard error, LCL = lower 95% confidence limit, UCL = upper 95% confidence limit.
(PDF)

S6 Table Output of the selected logistic regression model to assess litter detection probabilities in 50 × 2 m plots in relation to land cover type and distance to the nearest road in Steinkjer, Norway (H1c). The model including land-cover type and distance to the nearest road as additive terms outperformed all other candidate models (ΔAICc second ranked model = 3.43; i.e. the road model). β = estimate, se = standard error, z-value = test statistic, p-values < 0.05 are considered as statistically significant. For this analysis, we excluded land cover type factor levels ’Roadside’ and ‘Urban’ because of multicollinearity with distance to the nearest road.
(PDF)

S7 Table Output of the selected linear mixed effect regression model to assess litter fragment size in 50 × 2 m plots distributed across various land cover types in Steinkjer, Norway (H1d). The model including land-cover type outperformed the null model (ΔAICc = 9.78). β = estimate, se = standard error, t-value = test statistic. The random effects variance and standard deviation of ‘survey plot ID’ was 0.322 and 0.568, respectively.
(PDF)

S8 Table Predicted litter fragment sizes (cm) and their 95% confidence intervals for 50 × 2 m plots (N = 110, surveyed in early October 2020) distributed across various land cover types in Steinkjer, Norway (H1d). se = standard error, LCL = lower 95% confidence limit, UCL = upper 95% confidence limit.
(PDF)

S9 Table Cross table of the number of observed and expected (*in italic*) litter items for each material and land cover type. Litter was collected in 50 × 2 m plots (N = 110) surveyed in early October 2020 in Steinkjer, central Norway.
(PDF)

S1 Fig Residual vs. fitted values of the most parsimonious linear mixed effects regression model to assess if litter particle size varied among land cover types (H1d). The plot suggests that the model did not show clear heteroskedasticity.
(PDF)

S2 Fig Wordcloud of all registered litter items distributed by land cover type and in total. Litter items were registered in 110 sampling transects (50 x 2 m) distributed in a cultural landscape of central Norway during autumn 2020. Litter items are scaled in size relative to their abundance per land cover type and in total.
(JPG)

S1 Appendix (DOCX)

S1 Data (XLSX)

S2 Data (R)
