# Supplementary material for: Land cover type modulates the distribution of litter in a Nordic cultural landscape
Source: PLoS One. 2022 Nov 9;17(11):e0275463. doi: 10.1371/journal.pone.0275463 (PMC9645623; doi:10.1371/journal.pone.0275463)
Supplement: S1 Table — Checkmarks (✓) indicate inclusion of specific model terms in candidate models. GLM = generalized linear model, LMM = linear mixed effects model, df = degrees of freedom, AICc = Akaike Information Criterion corrected for small sample sized, ΔAICc = the difference in AICc value between compared to the candidate model with the lowest AICc value, AICcw = AICc model weight. (PDF) [file pone.0275463.s001.pdf]

**S1 Table.** Model selection diagnostics for the candidate models to evaluate H1a-d.

Checkmarks (✓) indicate inclusion of specific model terms in candidate models. GLM = generalized linear model, LMM = linear mixed effects model, df = degrees of freedom, AICc = Akaike Information Criterion corrected for small sample sized, ΔAICc = the difference in AICc value between compared to the candidate model with the lowest AICc value, AICcw = AICc model weight.

**H1a: litter detection varies probability across land cover types (binomial GLM)**

| *Candidate model* | *Land cover type* | *df* | *AICc* | *ΔAICc* | *AICcw* |
|---|---|---|---|---|---|
| Land cover model | ✓ | 8 | 136.2 | 0 | 1 |
| Null model | | 1 | 152.2 | 16.04 | 0 |

**H1b: litter abundance varies across land cover types (negative binomial GLM)**

| *Candidate model* | *Land cover type* | *df* | *AICc* | *ΔAICc* | *AICcw* |
|---|---|---|---|---|---|
| Land cover model | ✓ | 9 | 582.1 | 0 | 0.998 |
| Null model | | 2 | 594.7 | 12.51 | 0.002 |

**H1c: litter detection probabilities decrease with distance to the nearest road (binomial GLM)**

| *Candidate model* | *Distance to roads* | *Land cover type* | *Interaction* | *df* | *AICc* | *ΔAICc* | *AICcw* |
|---|---|---|---|---|---|---|---|
| Additive model | ✓ | ✓ | | 7 | 99.9 | 0 | 0.785 |
| Road model | ✓ | | | 2 | 103.3 | 3.43 | 0.141 |
| Interaction model | ✓ | ✓ | ✓ | 12 | 104.7 | 4.79 | 0.072 |
| Land cover model | | ✓ | | 6 | 112.6 | 12.70 | 0.001 |
| Null model | | | | 1 | 113.7 | 13.83 | 0.001 |

**H1d: litter particle size is varies across land cover types (LMM)**

| *Candidate model* | *Land cover type* | *df* | *AICc* | *ΔAICc* | *AICcw* |
|---|---|---|---|---|---|
| Land cover model | ✓ | 10 | 2686.4 | 0 | 0.993 |
| Null model | | 3 | 2696.2 | 9.78 | 0.007 |
