# Supplementary material for: Land cover type modulates the distribution of litter in a Nordic cultural landscape
Source: PLoS One. 2022 Nov 9;17(11):e0275463. doi: 10.1371/journal.pone.0275463 (PMC9645623; doi:10.1371/journal.pone.0275463)
Supplement: S2 Table — The model including land-cover type outperformed the null model (ΔAICc = 16.04). β = estimate, se = standard error, z-value = test statistic, p-values < 0.05 are considered as statistically significant. (PDF) [file pone.0275463.s002.pdf]

S2 Table. Output of the selected binomial logistic regression model to assess litter detection probabilities in 50 × 2 m plots (N = 110, surveyed in early October 2020) distributed across various land cover types in Steinkjer, Norway (H1a). The model including land-cover type outperformed the null model (ΔAICc = 16.04). β = estimate, se = standard error, z-value = test statistic, p-values < 0.05 are considered as statistically significant.

**Land cover type (factor levels)**	**β**	**se**	**z-value**	**p-value**
Agriculture	-0.511	0.516	-0.989	0.323
Beach	1.504	0.782	1.924	0.054
Edge	-1.204	0.658	-1.829	0.067
Forest	-0.588	0.558	-1.054	0.292
Lakeshore	0.588	0.558	1.054	0.292
River	-0.470	0.570	-0.824	0.410
Road	2.079	0.750	2.773	0.006
Urban	2.303	1.049	2.196	0.028
