# Supplementary material for: Land cover type modulates the distribution of litter in a Nordic cultural landscape
Source: PLoS One. 2022 Nov 9;17(11):e0275463. doi: 10.1371/journal.pone.0275463 (PMC9645623; doi:10.1371/journal.pone.0275463)
Supplement: S3 Table — se = standard error, LCL = lower 95% confidence limit, UCL = upper 95% confidence limit. (PDF) [file pone.0275463.s003.pdf]

S3 Table. Predicted litter detection probabilities and their 95% confidence intervals for 50 × 2 m plots (N = 110, surveyed in early October 2020) distributed across various land cover types in Steinkjer, Norway (H1a). se = standard error, LCL = lower 95% confidence limit, UCL = upper 95% confidence limit.

Land cover type (factor levels)	Prediction	LCL	UCL
Forest	0.357	0.157	0.624
Agriculture	0.375	0.179	0.623
Urban	0.909	0.562	0.987
River	0.385	0.170	0.656
Road	0.889	0.648	0.972
Edge	0.231	0.076	0.522
Lakeshore	0.643	0.376	0.843
Beach	0.818	0.493	0.954
