# Supplementary material for: Land cover type modulates the distribution of litter in a Nordic cultural landscape
Source: PLoS One. 2022 Nov 9;17(11):e0275463. doi: 10.1371/journal.pone.0275463 (PMC9645623; doi:10.1371/journal.pone.0275463)
Supplement: S4 Table — The model including land-cover type outperformed the null model (ΔAICc = 12.51). β = estimate, se = standard error, z-value = test statistic, p-values < 0.05 are considered as statistically significant. (PDF) [file pone.0275463.s004.pdf]

S4 Table. Output of the selected negative binomial logistic regression model to assess litter abundance in 50 × 2 m plots (N = 110, surveyed in early October 2020) distributed across various land cover types in Steinkjer, Norway (H1b). The model including land-cover type outperformed the null model (ΔAICc = 12.51). β = estimate, se = standard error, z-value = test statistic, p-values < 0.05 are considered as statistically significant.

**Land cover type (factor levels)**	**β**	**se**	**z-value**	**p-value**
Agriculture	-0.288	0.524	-0.549	0.583
Beach	3.018	0.531	5.684	< 0.001
Edge	0.143	0.549	0.261	0.794
Forest	1.168	0.490	2.382	0.017
Lakeshore	2.816	0.472	5.973	< 0.001
River	2.050	0.495	4.145	< 0.001
Road	2.494	0.417	5.976	< 0.001
Urban	2.009	0.538	3.732	< 0.001
