# Supplementary material for: Land cover type modulates the distribution of litter in a Nordic cultural landscape
Source: PLoS One. 2022 Nov 9;17(11):e0275463. doi: 10.1371/journal.pone.0275463 (PMC9645623; doi:10.1371/journal.pone.0275463)
Supplement: S5 Table — se = standard error, LCL = lower 95% confidence limit, UCL = upper 95% confidence limit. (PDF) [file pone.0275463.s005.pdf]

S5 Table. Predicted litter abundance and their 95% confidence intervals for 50 × 2 m plots (N = 110, surveyed in early October 2020) distributed across various land cover types in Steinkjer, Norway (H1b). se = standard error, LCL = lower 95% confidence limit, UCL = upper 95% confidence limit.

Land cover type (factor levels)	Prediction	LCL	UCL
Forest	3.214	1.230	8.400
Agriculture	0.750	0.269	2.093
Urban	7.455	2.600	21.407
River	7.769	2.947	20.485
Road	12.111	5.345	27.442
Edge	1.154	0.393	3.385
Lakeshore	16.714	6.634	42.113
Beach	20.455	7.225	57.910
