# Supplementary material for: Land cover type modulates the distribution of litter in a Nordic cultural landscape
Source: PLoS One. 2022 Nov 9;17(11):e0275463. doi: 10.1371/journal.pone.0275463 (PMC9645623; doi:10.1371/journal.pone.0275463)
Supplement: S6 Table — The model including land-cover type and distance to the nearest road as additive terms outperformed all other candidate models (ΔAICc second ranked model = 3.43; i.e. the road model). β = estimate, se = standard error, z-value = test statistic, p-values < 0.05 are considered as statistically significant. For this analysis, we excluded land cover type factor levels ’Roadside’ and ‘Urban’ because of multicollinearity with distance to the nearest road. (PDF) [file pone.0275463.s006.pdf]

S6 Table. Output of the selected logistic regression model to assess litter detection probabilities in 50 × 2 m plots in relation to land cover type and distance to the nearest road in Steinkjer, Norway (H1c). The model including land-cover type and distance to the nearest road as additive terms outperformed all other candidate models (ΔAICc second ranked model = 3.43; i.e. the road model). β = estimate, se = standard error, z-value = test statistic, p-values < 0.05 are considered as statistically significant. For this analysis, we excluded land cover type factor levels 'Roadside' and 'Urban' because of multicollinearity with distance to the nearest road.

**Model term**	**β**	**se**	**z-value**	**p-value**
Intercept	0.513	0.618	0.831	0.406
Distance to the nearest road	-0.009	0.003	-3.050	0.002
Land cover type - Beach	2.402	1.013	2.372	0.018
Land cover type - Edge	-1.079	0.876	-1.232	0.218
Land cover type - Forest	0.927	0.869	1.067	0.286
Land cover type - Lakeshore	1.233	0.840	1.469	0.142
Land cover type - River	0.864	0.869	0.994	0.320
