# Supplementary material for: Land cover type modulates the distribution of litter in a Nordic cultural landscape
Source: PLoS One. 2022 Nov 9;17(11):e0275463. doi: 10.1371/journal.pone.0275463 (PMC9645623; doi:10.1371/journal.pone.0275463)
Supplement: S7 Table — The model including land-cover type outperformed the null model (ΔAICc = 9.78). β = estimate, se = standard error, t-value = test statistic. The random effects variance and standard deviation of ‘survey plot ID’ was 0.322 and 0.568, respectively. (PDF) [file pone.0275463.s007.pdf]

S7 Table. Output of the selected linear mixed effect regression model to assess litter fragment size in 50 × 2 m plots distributed across various land cover types in Steinkjer, Norway (H1d). The model including land-cover type outperformed the null model (ΔAICc = 9.78). β = estimate, se = standard error, t-value = test statistic. The random effects variance and standard deviation of 'survey plot ID' was 0.322 and 0.568, respectively.

**Land cover type (factor levels)**	**β**	**se**	**t- value**
Intercept - Agriculture	2.892	0.374	7.73
Beach	-0.562	0.447	-1.258
Edge	0.404	0.561	0.721
Forest	-0.689	0.501	-1.377
Lakeshore	-0.23	0.427	-0.539
River	-0.35	0.493	-0.71
Road	-0.873	0.413	-2.118
Urban	-1.338	0.437	-3.066
