# Supplementary material for: Land cover type modulates the distribution of litter in a Nordic cultural landscape
Source: PLoS One. 2022 Nov 9;17(11):e0275463. doi: 10.1371/journal.pone.0275463 (PMC9645623; doi:10.1371/journal.pone.0275463)
Supplement: S8 Table — se = standard error, LCL = lower 95% confidence limit, UCL = upper 95% confidence limit. (PDF) [file pone.0275463.s008.pdf]

S8 Table. Predicted litter fragment sizes (cm) and their 95% confidence intervals for 50 × 2 m plots (N = 110, surveyed in early October 2020) distributed across various land cover types in Steinkjer, Norway (H1d). se = standard error, LCL = lower 95% confidence limit, UCL = upper 95% confidence limit.

Land cover type (factor levels)	Prediction	LCL	UCL
Forest	9.049	4.713	17.376
Agriculture	18.032	8.661	37.542
Urban	4.729	3.044	7.348
River	12.709	6.784	23.809
Road	7.529	5.357	10.581
Edge	27.014	11.920	61.222
Lakeshore	14.321	9.557	21.462
Beach	10.277	6.364	16.598
