# Supplementary material for: Land cover type modulates the distribution of litter in a Nordic cultural landscape
Source: PLoS One. 2022 Nov 9;17(11):e0275463. doi: 10.1371/journal.pone.0275463 (PMC9645623; doi:10.1371/journal.pone.0275463)
Supplement: S9 Table — Litter was collected in 50 × 2 m plots (N = 110) surveyed in early October 2020 in Steinkjer, central Norway. (PDF) [file pone.0275463.s009.pdf]

S9 Table. Cross table of the number of observed and expected (*in italic*) litter items for each material and land cover type. Garbage was collected in 50 × 2 m plots (N = 110) surveyed in early October 2020 in Steinkjer, central Norway.

	Metal	Other	Paper	Plastic	Row total
**Agriculture**	1	1	0	10	12
	1.082	*0.528*	*0.747*	*9.644*	
**Beach**	3	13	2	207	225
	20.279	*9.898*	*14.002*	*180.821*	
**Edge**	2	0	3	10	15
	1.352	*0.660*	*0.933*	*12.055*	
**Forest**	0	1	1	43	45
	4.056	*1.980*	*2.800*	*36.164*	
**Lakeshore**	15	7	3	209	234
	21.090	*10.294*	*14.562*	*188.054*	
**River**	36	5	1	59	101
	9.103	*4.443*	*6.285*	*81.168*	
**Road**	19	13	29	157	218
	19.648	*9.590*	*13.567*	*175.195*	
**Urban**	8	1	19	54	82
	7.391	*3.607*	*5.103*	*65.899*	
**Column total**	84	41	58	749	932
