# Supplementary material for: Land cover type modulates the distribution of litter in a Nordic cultural landscape
Source: PLoS One. 2022 Nov 9;17(11):e0275463. doi: 10.1371/journal.pone.0275463 (PMC9645623; doi:10.1371/journal.pone.0275463)

S1 Fig. Residual vs. fitted values of the most parsimonious linear mixed effects regression model to assess if litter particle size varied among land cover types (H1d). The plot suggests that the model did not show clear heteroskedasticity.

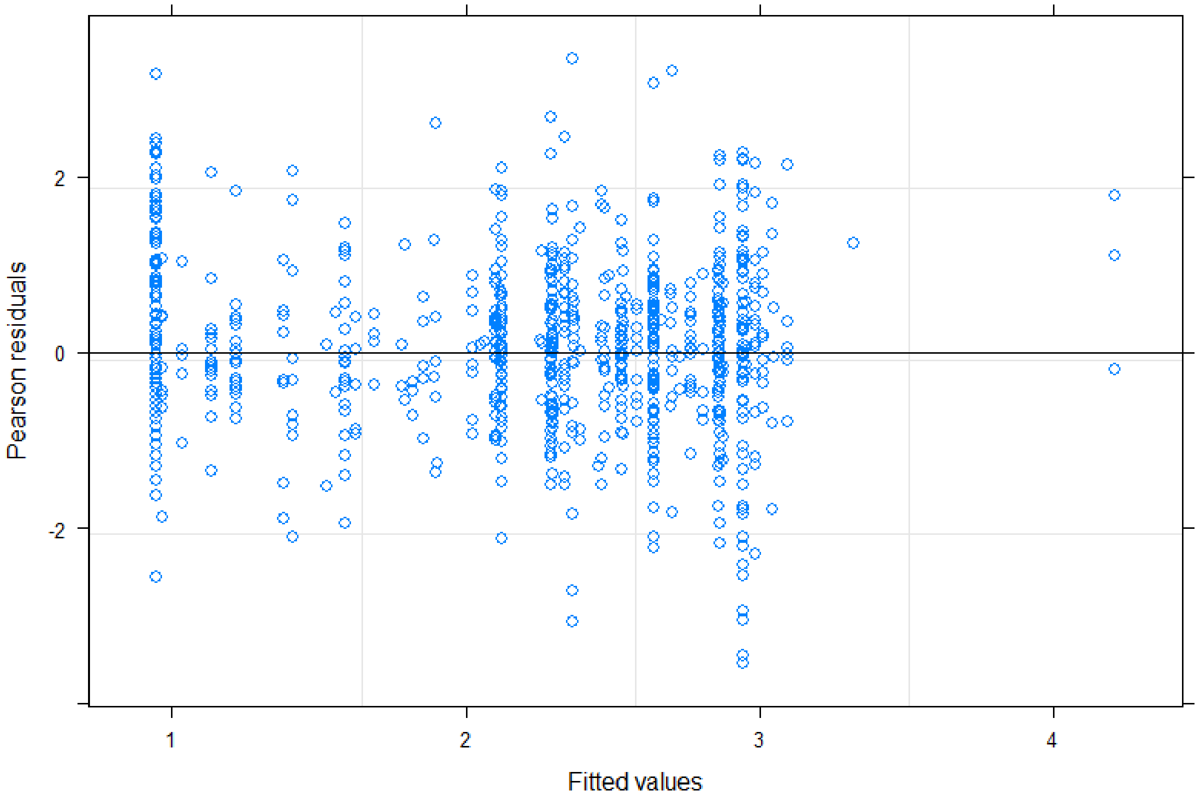

Supplement: S1 Fig — The plot suggests that the model did not show clear heteroskedasticity. (PDF) [file pone.0275463.s010.pdf]
