# Supplementary figures and images for: Land cover type modulates the distribution of litter in a Nordic cultural landscape
Source: PLoS One. 2022 Nov 9;17(11):e0275463. doi: 10.1371/journal.pone.0275463 (PMC9645623; doi:10.1371/journal.pone.0275463)

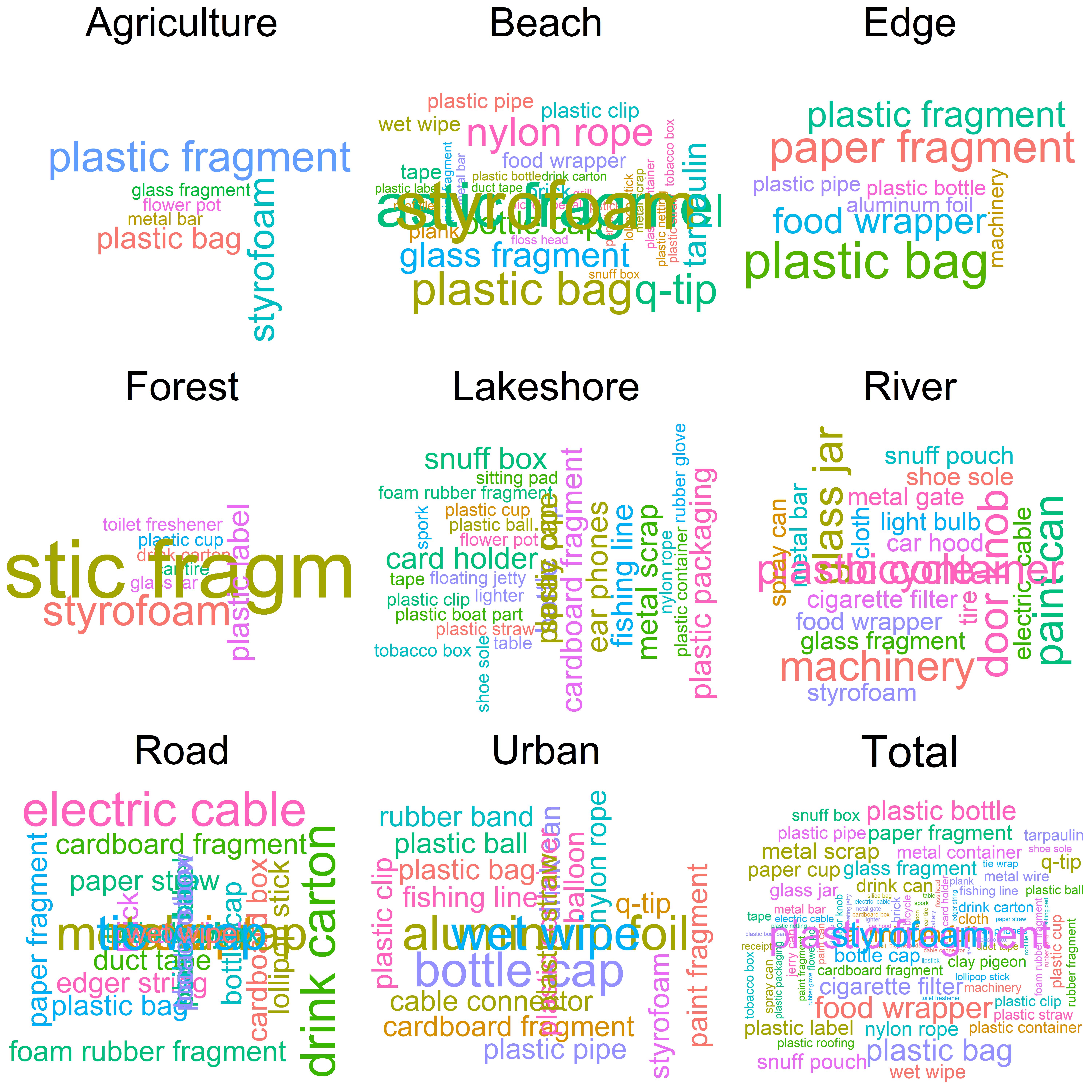

Supplement: S2 Fig — Litter items were registered in 110 sampling transects (50 x 2 m) distributed in a cultural landscape of central Norway during autumn 2020. Litter items are scaled in size relative to their abundance per land cover type and in total. (JPG) [file pone.0275463.s011.jpg]
