# Supplementary material for: Land cover type modulates the distribution of litter in a Nordic cultural landscape
Source: PLoS One. 2022 Nov 9;17(11):e0275463. doi: 10.1371/journal.pone.0275463 (PMC9645623; doi:10.1371/journal.pone.0275463)
Supplement: S1 Appendix — (DOCX) [file pone.0275463.s012.docx]

Supporting information – R files

**S1A Appendix: Geoprocessing code.** <https://osf.io/ygfme/> (SOM_Geoprocessing)

**S1B Appendix: Data.** <https://osf.io/ygfme/> (Nord_litterProject_2020.xlsx)

**S1C Appendix: R code.** <https://osf.io/ygfme/> (Garbage_script.R)
